# Dedicated MRI staging versus surgical staging of peritoneal metastases in colorectal cancer patients considered for CRS-HIPEC; the DISCO randomized multicenter trial

**DOI:** 10.1186/s12885-021-08168-x

**Published:** 2021-04-26

**Authors:** M. P. Engbersen, C. J. V. Rijsemus, J. Nederend, A. G. J. Aalbers, I. H. J. T. de Hingh, V. Retel, D. M. J. Lambregts, E. J. R. J. Van der Hoeven, D. Boerma, M. J. Wiezer, M. De Vries, E. V. E. Madsen, A. R. M. Brandt-Kerkhof, S. Van Koeverden, P. R. De Reuver, R. G. H. Beets-Tan, N. F. M. Kok, M. J. Lahaye

**Affiliations:** 1 Department of Radiology, Netherlands Cancer Institute, Plesmanlaan 121, 1066 CX Amsterdam, The Netherlands; 2 GROW, School for Oncology and Developmental Biology, Maastricht University Medical Center, Maastricht, The Netherlands; 3 Department of Surgery, Netherlands Cancer Institute, Plesmanlaan 121, 1066 CX Amsterdam, The Netherlands; 4 Department of Radiology, Catharina Hospital, Michelangelolaan 2, 5623 EJ Eindhoven, The Netherlands; 5 Department of Surgery, Catharina Hospital, Michelangelolaan 2, 5623 EJ Eindhoven, The Netherlands; 6 Department of Psychosocial research and Epidemiology, Netherlands Cancer Institute, Plesmanlaan 121, 1066 CX Amsterdam, The Netherlands; 7 Department Health Technology and Services Research (HTSR), University of Twente, Drienerlolaan 5, Enschede, The Netherlands; 8 Department of Radiology, St. Antonius Hospital, Soestwetering 1, 3543 AZ Utrecht, The Netherlands; 9 Department of Surgery, St. Antonius Hospital, Soestwetering 1, 3543 AZ Utrecht, The Netherlands; 10 Department of Radiology, Erasmus University Medical Center, Dr. Molewaterplein 40, 3015 GD Rotterdam, The Netherlands; 11 Department of Surgery, Erasmus University Medical Center, Dr. Molewaterplein 40, 3015 GD Rotterdam, The Netherlands; 12 Department of Radiology, Radboud University Medical Center, P.O. Box 9101, 6500 HB Nijmegen, the Netherlands; 13 Department of Surgery, Radboud University Medical Center, P.O. Box 9101, 6500 HB Nijmegen, the Netherlands

**Keywords:** Colorectal peritoneal metastases, CRS-HIPEC, Surgical staging, MRI, RCT

## Abstract

**Background:**

Selecting patients with peritoneal metastases from colorectal cancer (CRCPM) who might benefit from cytoreductive surgery followed by hyperthermic intraperitoneal chemotherapy (CRS-HIPEC) is challenging. Computed tomography generally underestimates the peritoneal tumor load. Diagnostic laparoscopy is often used to determine whether patients are amenable for surgery. Magnetic resonance imaging (MRI) has shown to be accurate in predicting completeness of CRS. The aim of this study is to determine whether MRI can effectively reduce the need for surgical staging.

**Methods:**

The study is designed as a multicenter randomized controlled trial (RCT) of colorectal cancer patients who are deemed eligible for CRS-HIPEC after conventional CT staging. Patients are randomly assigned to either MRI based staging (arm A) or to standard surgical staging with or without laparoscopy (arm B). In arm A, MRI assessment will determine whether patients are eligible for CRS-HIPEC. In borderline cases, an additional diagnostic laparoscopy is advised. The primary outcome is the number of unnecessary surgical procedures in both arms defined as: all surgeries in patients with definitely inoperable disease (PCI > 24) or explorative surgeries in patients with limited disease (PCI < 15). Secondary outcomes include correlations between surgical findings and MRI findings, cost-effectiveness, and quality of life (QOL) analysis.

**Conclusion:**

This randomized trial determines whether MRI can effectively replace surgical staging in patients with CRCPM considered for CRS-HIPEC.

**Trial registration:**

Registered in the clinical trials registry of U.S. National Library of Medicine under NCT04231175.

## Background

Peritoneal metastatic disease is the dissemination of cancer within the abdominal cavity. It is the second leading cause of death in patients with colorectal cancer (CRC). 10.6% of CRC patients present with peritoneal metastases at diagnosis or will develop peritoneal metastases at a later stage [1]. Selected patients benefit from cytoreductive surgery (CRS) followed by hyperthermic intraperitoneal chemotherapy (HIPEC). CRS-HIPEC is potentially curative and has shown to improve median survival in patients with limited disease [2–4]. Macroscopically complete resection of all metastatic lesions is vital to obtain survival gain [5]. Currently, assessment of the extent and localization of peritoneal metastases, to determine whether a complete CRS is feasible, is ultimately performed during laparotomy. Open-close or incomplete procedures have been reported to occur in 20 to 40% of cases [6–11]. However, best practice would be to avoid futile, invasive and costly surgical procedures in patients for whom CRS-HIPEC is not feasible and/or unlikely to improve survival. Laparoscopic assessment is a less invasive and effective tool to reduce futile laparotomies [9]. However, tumor mass or adhesions may prevent laparoscopic evaluation of all regions of the peritoneal cavity. Incomplete laparoscopic staging has been reported to occur in around 23% of patients [6]. Laparoscopy is considered minimally invasive but a risk of intraoperative complications and postoperative morbidity presides. Hence, a non-invasive pre-operative tool is needed to better select patients eligible for CRS-HIPEC.

CT is the preferred first-line imaging tool for the detection of peritoneal metastases but falls short when used to assess the total peritoneal tumor burden [12–16]. Magnetic resonance imaging (MRI), with diffusion-weighted imaging (DWI), has shown to predict the extent of disease accurately (sensitivity of 0.80–0.98 and a specificity 0.85–0.93% [17, 18]) and to be a valuable adjunct to decide whether complete cytoreduction can be achieved [13, 15, 17]. DWI-MRI had also shown to be superior for detecting liver metastases [19, 20]. However, research towards the value of MRI for patients with colorectal peritoneal metastases has been limited to cohort studies in a retrospective or observational setting.

### Objective

The primary objective of this study is to determine whether additional preoperative dedicated MRI staging to can reduce the number of futile (exploratory) surgeries in patients with CRC considered for CRS-HIPEC as compared to the standard of care of CT alone. An unnecessary surgical procedure is defined as any non-therapeutic laparotomy or diagnostic laparoscopy in patients, with exception of patients with borderline operable disease (15<PCI> 24).

## Methods/design

**Fig. 1:**
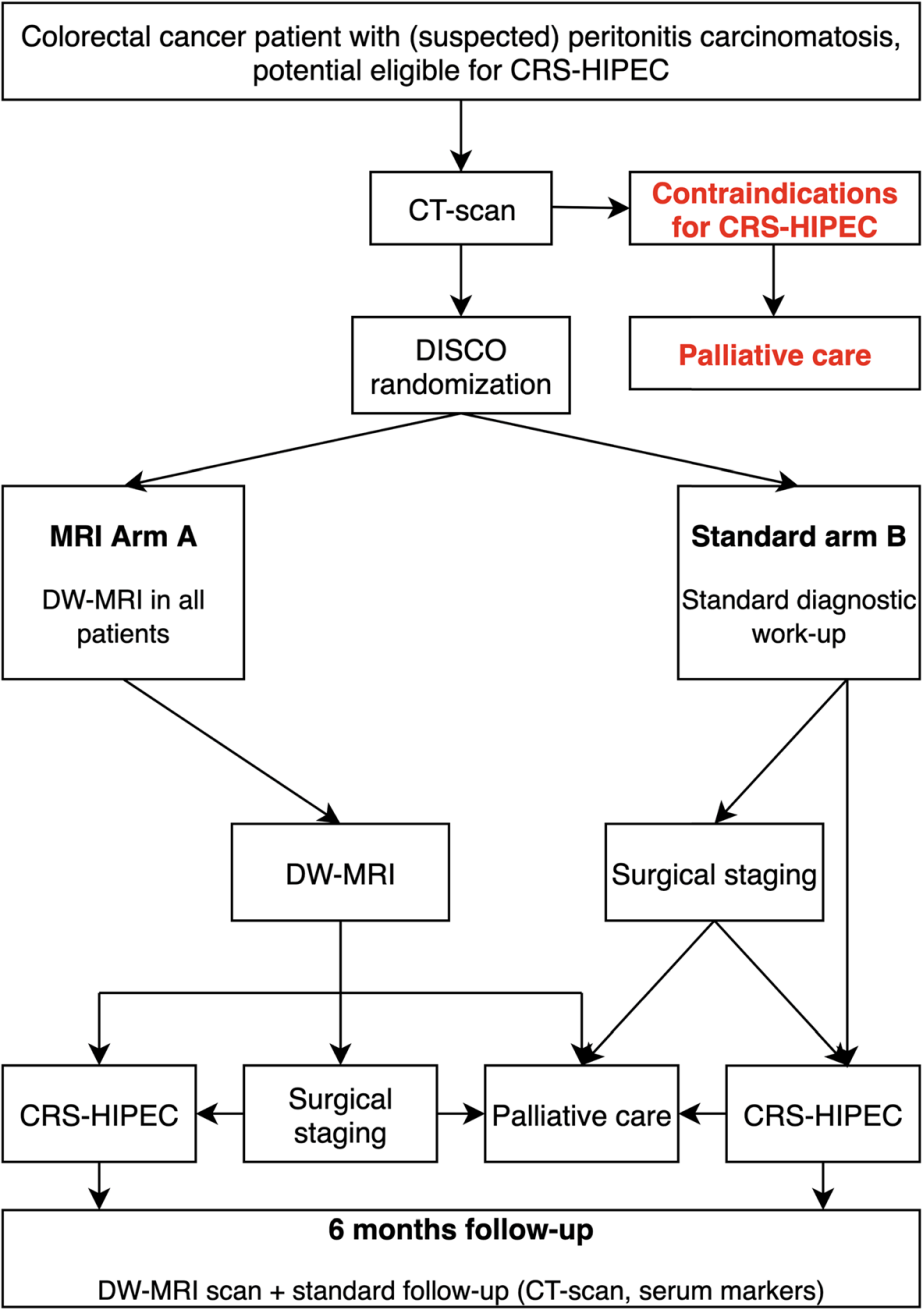
Flow diagram of DISCO trial

This investigator-initiated, multi-center, randomized controlled trial is performed in Dutch HIPEC centers since September 2019. The study is conducted according to the principles of the Declaration of Helsinki and in accordance with the Medical Research Involving Human Subjects Act (WMO) and was approved by the medical ethics committee at the Netherlands Cancer Institute (date: 13-09-2019; ref. no.: NL70045.031.19) and is retrospectively registered in the clinical trials registry of U.S. National Library of Medicine under NCT04231175 (https://clinicaltrials.gov/ct2/show/NCT04231175). Patients suspected of having CRC peritoneal metastases and considered for CRS-HIPEC, are eligible for inclusion. Patients are randomly assigned to one of two diagnostic arms towards CRS-HIPEC. A trial flow diagram is presented in Fig. 1.

### Eligibility criteria

In order to be eligible to participate in this study, a subject must meet all of the following criteria:

- Patients with suspicion of CRCPM and considered for CRS-HIPEC after CT imaging
- Age ≥ 18 years
- Written and signed informed consent
- Performance status of WHO 0–2
- Able and willing to drink 1 l of pineapple or blueberry juice (required as a preparatory step for the dedicated MRI)

A potential subject who meets any of the following criteria will be excluded from participation in this study:

- Patients with contraindications for MRI
- Patients with clinical contraindications for CRS-HIPEC, such as but not limited to inadequate organ or hematological function, significant medical history, active pregnancy and lactation, or old age
- Patients with radiological contra-indications for CRS-HIPEC observed on CT thorax/abdomen
- Patients with concurrent or history of other malignancy within ≤5 years prior to CRCPM diagnosis

### Patient recruitment and randomization

Eligible patients are identified and informed about the study by their treating physician at the participating center. Patients are included after written informed consent. Patients are randomly assigned to an experimental arm (A) or a control arm (B). The random assignment is performed by dedicated medical data managers or a research nurse using specific clinical trial management software (ALEA clinical, Abcoude, The Netherlands). A random block randomization is used and stratified per participating center. Blinding is not possible with this trial set-up.

### Study interventions

Patients in Arm A receive a dedicated 3 T MRI scan, which includes T2 weighted, T1 weighted, diffusion weighted and non-enhanced + gadolinium enhanced fat-suppressed T1 weighted imaging of the pelvis and abdomen (and thorax on DWI), as previously described [15]. Patients are asked to drink one liter of pineapple or blueberry at least 1 h before the MRI scan. This minimizes the signal from the intraluminal content on DWI and bowel movement, respectively. An antispasmolitic drug is administered intravenously to optimize bowel evaluation. The MRI protocol is specified in Table 1. Based on the findings of the MRI scan, the radiologist advises whether patients are to be allocated to one of the following diagnostic/treatment options:

- CRS-HIPEC is feasible; perform CRS-HIPEC
  - ○ Peritoneal cancer index estimated on MRI (MRI-PCI) < 15 and no non-peritoneal lesions (that contra-indicate CRS-HIPEC)
- CRS-HIPEC might be feasible; consider diagnostic laparoscopy (DLS) to determine whether CRS-HIPEC is feasible and justifiable
  - ○ MRI-PCI between 15 and 24 and no non-peritoneal lesions (that contra-indicate CRS-HIPEC)
- CRS-HIPEC is not feasible; chemotherapy or best supportive care
  - ○ MRI-PCI > 24 and/or detection of non-peritoneal lesions (that contra-indicate CRS-HIPEC)

**Table 1:** Dedicated MRI protocol for peritoneal surface malignancies

	Diffusion weighted	T2 weighted		T1 weighted		
				Pre-contrast	Post-contrast	Post contrast
Pulse sequence	SS EPI b0, b1000	SS TSE	SS TSE	mDixon 3D FFE	mDixon 3D FFE	mDixon 3D FFE
Imaging plane	Transversal	Transversal	Coronal	Transversal	Transversal	Coronal
Anatomical area	Thorax, abdomen, pelvis	Abdomino-pelvic	Thorax, abdomen, pelvis	Abdomino-pelvic	Abdomino-pelvic	Abdomino-pelvic
Respiration	Free-breathing	Free-breathing	Free-breathing	Free-breathing	Free-breathing	Free-breathing
Fat supression	SPIR	–	–	SPAIR	SPAIR	SPAIR
TR/TE (ms)	Shortest	1500/87	1500/87	Shortest	Shortest	Shortest
NSA	1	1	1	1	1	1
Slice thickness (mm)	5	4	6	5	5	5
Gap (mm)	0.5	0.4	0.6	−2.5	−2.5	−2.5

In the Netherlands, patients are eligible for CRS-HIPEC if the PCI does not exceed 20 at surgical exploration [21]. All participating centers perform CRS-HIPEC procedures under the Dutch HIPEC protocol, as described by Kuijpers and colleagues [21]. With this policy in mind, the cut-off points for MRI-assessment have been determined using preliminary data of the initiating center [15]. The MRI-PCI cut-off of 15 ensures targeted use of DLS and the cut-off of 24 prevents the false omission of patients for whom CRS-HIPEC is feasible. If a specific disease localization is found which could contraindicate CRS-HIPEC, i.e. duodenal involvement, DLS may still be performed at the discretion of the multidisciplinary team (MDT). Surgical procedures must be performed within 4 weeks after the MRI.

In arm B, patients undergo the standard diagnostic work-up followed by a DLS at the discretion of the MDT, as determined before randomization. For the control group no study specific guidelines for DLS use have been determined, centers are expected to continue implementing DLS according to their own current policies. The rate of laparoscopy use may therefore vary between centers.

All patients will be asked to fill in two QOL questionnaires (EORTC-C30 and EQ5D5L) at baseline (randomization), at 3 months, and 6 months. At 6 months after CRS-HIPEC, all patients undergo a follow-up of CT, MRI, serum biomarkers (carcinoembryonic antigen or CEA, cancer antigen 125 or CA 125, and cancer antigen 19.9 or CA 19.9), and physical examination to screen for early disease recurrence in the peritoneum, liver, or elsewhere. This will also serve as an extra quality check of diagnostic and surgical outcome. It is expected that a residual peritoneal or non-peritoneal metastasis will have become apparent at 6 months on MRI. If residual disease of early recurrence is observed, treatment is at the discretion of the local MDT. The trial schedule is summarized in Table 2.

**Table 2:** Schedule of enrolment, interventions, and assessments

STUDY PERIOD						
	Enrolment	Allocation	Diagnostic Work-up	Treatment	Follow-up	
TIMEPOINT^b^	***-T***_***1***_	T_**0**_	T_**1**_	T_**1**_	***T***_***3***_	***T***_***6***_
**ENROLMENT:**						
**Eligibility screen**	X					
**Informed consent**	X					
**Allocation**		X				
**INTERVENTIONS**						
***MRI***			A			X^b^
***Diagnostic laparoscopy***			B^a^/A^a^			
***CRS-HIPEC***				A^a^/B^a^		
**ASSESSMENTS**						
***QUALY forms***		X			X	X

X applicable to all patients; A applicable to those allocated to experimental Arm A; B applicable to those allocated to control arm B

^a^ only applicable if called for by previous diagnostics

^b^ only applicable if R1 resection was achieved at CRS-HIPEC

### MRI evaluation

All study MRIs will be evaluated by an abdominal radiologist with experience in DWI of the participating center and by experienced abdominal radiologists of the trial-initiating center with extensive experience with the study protocol dedicated to peritoneal metastasis evaluation, independently. The findings of the radiologists then will be shared and discussed before the MDT meeting. In case of discrepant findings, the findings from the trial-initiating center’s radiologist are leading. Radiologists of the participating centers will undergo training in the study specific MRI evaluations, followed by on-the-job training with continued feedback of the trial-initiating center.

Criteria used to establish the presence of peritoneal disease on MRI include the presence of high signal on DWI with a corresponding soft tissue lesion of peritoneal thickening on T2 weighted or contrast enhanced T1 weighted images, as previously described [15].

### Data collection

Basic clinical information is collected, including patient history, primary tumor location, tumor histology, previous surgical procedures, chemotherapy and/or radiotherapy history, and serum biomarkers. For each abdominal assessment preformed to assess eligibility for CRS-HIPEC (CT, MRI, laparoscopy, and laparotomy), a case report form (CRF) is filled out including the same parameters for each assessment, where appropriate. These parameters include the PCI, the presence of organ infiltration, the presence and pattern of miliary spread, presence of non-PM (such as liver and lymph node metastases) and diaphragmatic involvement. Parameters specific to radiological assessments (CT, MRI) include scan quality, scan completion, presence of extra abdominal metastases (retroperitoneum, chest), and presence of pleural effusion. For each radiological finding, the reader will indicate whether the feature is present (yes/no) and how certain he or she is of that decision (not certain/ quite certain/ certain). Parameters specific to surgical assessment include extent of adhesiolysis and accessibility. Surgical findings are considered the reference standard. No confidence level will be applied to those parameters. For the laparotomies that proceed to CRS-HIPEC, a separate CRF registers which resections, excisions, and/or extirpations have been performed, as well as the completeness of cytoreduction (resection status). All data is collected by the local research team, consisting of at least one radiologist, one surgeon, and a research nurse and/or medical data manager. The pseudo-anonymized data is entered and stored with the same clinical trial management software used for randomization (ALEA clinical, Abcoude, The Netherlands) in an ISO 27001 certified hosting facility in the Netherlands.

### Outcome measures

The primary outcome measure is a composite endpoint of futile surgical explorations. A futile surgical exploration is defined as: all surgeries in patients with definitely inoperable disease (PCI > 24) or explorative surgeries in patients with limited disease (PCI < 15).

The secondary outcomes include:

- number of incomplete CRS-HIPECs (R2a/ R2b/ open-close),
- number of residual disease events or early recurrences at 6 months after a complete resection at CRS-HIPEC,
- number of non-peritoneal metastases,
- diagnostic performance of MRI and diagnostic laparoscopy to predict whether complete CRS-HIPEC is feasible,
- correlations between MRI findings and surgical findings,
- incremental cost-effectiveness,
- Patient reported QOL between study arms.

### Costs and patient reported quality of life measures

Direct medical costs include both the immediate medical costs such as diagnostic tests and initial treatments (including side-effects), as well as later treatments. Direct costs incorporated in the current study include costs of standard diagnostic staging (CT+/−laparoscopy), CRS-HIPEC and treatment of recurrences, follow-up visits and palliative care. Resource use data will be collected from the hospital’s administrative database. Where appropriate, Dutch guidelines for costing studies will be used in applying tariffs to units of resource use [22].

For the health related quality of life (QoL), the EORTC-C30 questionnaire will be used [23]. For cost-effectiveness purposes, utilities will be measured, in order to derive quality-adjusted-life-years (QALYs). The utilities will reflect the preferences of society for length of life versus quality of life. The utilities will be derived from the trial by means of the EQ5D5L questionnaire [24]. The questionnaires are given to patients at baseline (T0), 3 months after the diagnostic staging and treatment (T1), and after 6 months (T2).

### Statistical analysis

#### Sample size calculation

Power calculations are based on an expected reduction of futile surgical explorations from 30 to 15% by MRI staging. With a two-sample two-sided test of binomial proportions and an α of 0.05, a ß of 0.20, and an expected drop-out rate of 20%, 272 patients are to be included in total.

### Data analysis

The observed difference in preventable surgical explorations between trial arms will be tested with a McNemar’s chi-squared test, as well as the difference in incomplete CRS-HIPECs, non-peritoneal findings before surgery, and early recurrences or residual disease visible at first follow-up (6 months). Progression free survival will be analyzed by binomial logical regression analysis. Progression free survival is defined as the time in days from complete CRS-HIPEC to recurrence or progression of disease as determined by the multidisciplinary team meeting or death of any cause. Correlations between MRI staging and surgical staging will be evaluated by intraclass correlation coefficients (ICC), Cohen’s kappa, and Cohen’s weighted kappa, where appropriate. Diagnostic performance of MRI and surgical staging to predict complete CRS will be compared by confusion matrix analysis and receiver operator characteristic analysis. Differences in predictive values will be tested with McNemar’s test and the DeLong test, where appropriate.

### Cost-effectiveness analysis

A cost-effectiveness Analysis will be performed with incremental costs per Quality Adjusted Life Year gained as outcome for patients with PM undergoing MRI versus usual diagnostic work-up for selection for CRS-HIPEC. A Markov model will be constructed, a lifelong time horizon with 3 months cycle length will be used, and a societal perspective from the Netherlands will be adopted. Future costs and effects will be discounted to their present value by a rate of 4 and 1.5% per year respectively, according to Dutch guidelines [25]. The results of the probabilistic sensitivity analysis will be illustrated in a cost-effectiveness plane, and cost-effectiveness acceptability curves (CEACs) will show decision uncertainty. CEACs show the probability that a pathway has the highest net monetary benefit, and thus is deemed cost-effective, for a range of Willingness to Pay values for one additional QALY, also referred as the ceiling ratio. In this analysis, we will use the Dutch (informal) ceiling ratio of €80,000 per QALY [26].

## Discussion

Selected CRC patients with limited PM can benefit from CRS-HIPEC with a chance of a significantly prolonged survival and in some cases even cure. Currently, the final clinical decision of whether complete resection at CRS-HIPEC is feasible is often determined during surgery. Rehabilitation from open-close surgery is not in the best interest of a palliative patient and may delay palliative treatment or inclusion in trials. Two trials are ongoing in the Netherlands exploring novel palliative strategies for patients with extensive CRCPM, namely the use of concomitant intraperitoneal and systemic chemotherapy (INTERACT) and repetitive electrostatic pressurized intraperitoneal aerosol chemotherapy with oxaliplatin (ePIPAC) [27, 28].

Twenty-three percent of candidates for the PRODIGE-7 trial were excluded at laparotomy due to the extent of PM, liver metastases or non-resectable disease [11]. Other studies have reported incomplete CRS rates of around 20–40%, when selecting patients suitable for CRS-HIPEC with CT [6, 7, 10]. To help reduce the incomplete CRS rate, laparoscopy can be used to better assess the feasibility of complete cytoreduction. Improvements of 7–15% in the number of incomplete CRS-HIPECs have been reported after clinical implementation of laparoscopy [10, 29]. A cohort study researching the clinical impact of implementing DLS into the diagnostic work-up reported that in 35.2% not all abdominopelvic regions were visualized at DLS [10]. PPVs for predicting complete cytoreduction have been reported of 0.63–0.97 with DLS [6, 8–10, 30]. Sensitivities and specificities of 0.80–0.98 and 0.85–0.93 have been reported with use of MRI at region-wise analysis in two cohort studies of 27 and 60 patients [17, 18], from with a PPV of 0.90 could be achieved for predicting an operable extent of disease [17]. This was in concordance with the findings of Van’t Sant and colleagues who reported a PPV of 0.89 finding an operable extent of disease in a cohort study of 49 patients [15]. MRI can give information about peritoneal involvement in all regions, even those that might not be accessible with DLS due to adhesions, tumor, or anatomical limitations (such as diaphragmatic dorsal area and gastrosplenic ligament). MRI staging may therefore aid operative planning. MRI is furthermore non-invasive and much less costly than a diagnostic laparoscopy.

A limitation of MRI in the work up of CRC patients with PM is the lack of histopathological proof of identified lesions. However, we expect that in most cases patients will have lesions that are accessible by biopsy or present with proven PM during a previous surgical procedure. If this is not the case, a DLS may still be necessary. Patients with severe claustrophobia as well as patients with metal implants non-compatible with MRI are not eligible for MRI assessment. Policy differences between hospitals may lead to differences in laparoscopy rates and open-close rates in the control arm. Assuming that the rate of DLS is inversely related to the incomplete and open-close rate, the potential benefit of additional MRI staging could nonetheless become apparent. Furthermore, the benefit of Oxaliplatin-based HIPEC in the combined CRS-HIPEC procedure has recently come under debate following the results of the PRODIGE-7 trial [11]. However, this study reinforced the notion that complete cytoreduction is the most determinant factor of survival, a finding that underlines how important it is to safely and effectively select those patients for whom a complete CRS is feasible. Therefor our trial would continue to be relevant to select patients for CRS, alone or CRS in combination with any perioperative therapy.

If MRI staging effectively reduces the number of surgical explorations in CRS-HIPEC candidates, patients with PM from CRC will be exposed to a less invasive and more effective triaging system.

## Data Availability

The datasets used and/or analysed during the current study are available from the corresponding author on reasonable request.

## Abbreviations

CA: 125 cancer antigen 125
CA: 19.9 cancer antigen 19.9
CEA: Carcinoembryonic antigen
CEAC: Cost-effectiveness acceptability curve
CRC: Colorectal cancer
CRCPM: Colorectal peritoneal metastases
CRS: Cytoreductive surgery
CT: Computed tomography
DLS: Diagnostic laparoscopy
DWI: Diffusion weighted imaging
ePIPAC: Electrostatic pressurized intraperitoneal aerosol chemotherapy
HIPEC: Hyperthermic intraperitoneal chemotherapy
ICC: Intraclass correlation coefficient
MDT: Multidisciplinary team
MRI: Magnetic resonance imaging
PCI: Peritoneal cancer index
PM: Peritoneal metastases
RCT: Randomized controlled trial
WHO: World health organization
QOL: Quality of life
QUALY: Quality adjusted life years
